# Progestin Resistance and Corresponding Management of Abnormal Endometrial Hyperplasia and Endometrial Carcinoma

**DOI:** 10.3390/cancers14246210

**Published:** 2022-12-15

**Authors:** Mu Lv, Peiqin Chen, Mingzhu Bai, Yan Huang, Linxia Li, Youji Feng, Hong Liao, Wenxin Zheng, Xiaojun Chen, Zhenbo Zhang

**Affiliations:** 1 Reproductive Medicine Center, Department of Obstetrics and Gynecology, Tongji Hospital, School of Medicine, Tongji University, Shanghai 200065, China; 2 Department of Obstetrics and Gynecology, Shanghai General Hospital, Shanghai Jiao Tong University School of Medicine, Shanghai 200080, China; 3 Department of Obstetrics and Gynecology, The International Peace Maternity & Child Health Hospital of China Welfare Institute, Shanghai Jiao Tong University School of Medicine, Shanghai 200030, China; 4 Reproductive Medicine Center, Maternal and Child Health Hospital in Xuzhou, Xuzhou 215002, China; 5 Department of Gynecologic Oncology, Fudan University Shanghai Cancer Center, 270 Dong-an Road, Shanghai 200032, China; 6 Department of Obstetrics and Gynecology, Seventh People’s Hospital of Shanghai University of Traditional Chinese Medicine, 358 Datong Road, Shanghai 200137, China; 7 Department of Clinical Laboratory Medicine, Shanghai First Maternity and Infant Hospital, Tongji University School of Medicine, Shanghai 200040, China; 8 Department of Pathology, University of Texas Southwestern Medical Center, Dallas, TX 75390, USA; 9 Department of Obstetrics and Gynecology, University of Texas Southwestern Medical Center, Dallas, TX 75390, USA; 10 Department of Gynecology, Obstetrics and Gynecology Hospital of Fudan University, Shanghai 200090, China

**Keywords:** progestin, progestin resistance, endometrial hyperplasia, endometrial carcinoma

## Abstract

**Simple Summary:**

Patients with endometrial hyperplasia (EH) and endometrial cancer (EC) often fail conservative treatment due to progestin resistance. This article reviews the therapeutic role of progestin in EH and EC as well as the mechanisms of progestin resistance, and systematically expounds potential therapeutic approaches to overcome progestin resistance, thus providing theoretical support for effective treatment strategies.

**Abstract:**

With a younger tendency in morbidity age, endometrial cancer (EC) incidence has grown year after year. Worse, even more commonly occurring is endometrial hyperplasia (EH), which is a precancerous endometrial proliferation. For young women with early EC and EH who want to preserve fertility, progestin therapy has been utilized as a routine fertility-preserving treatment approach. Nevertheless, progestin medication failure in some patients is mostly due to progestin resistance and side effects. In order to further analyze the potential mechanisms of progestin resistance in EH and EC, to provide theoretical support for effective therapeutic strategies, and to lay the groundwork for searching novel treatment approaches, this article reviews the current therapeutic effects of progestin in EH and EC, as well as the mechanisms and molecular biomarkers of progestin resistance, and systematically expounds on the potential therapeutic methods to overcome progestin resistance.

## 1. Introduction

Endometrial hyperplasia (EH) is an abnormal proliferation of epithelial cells and glands. If the hyperplastic endometrium persists, it may advance to atypical endometrial hyperplasia (AEH) or endometrioid intraepithelial neoplasia (EIN) [1]. AEH and EIN are precursors of endometrial cancer (EC). EC is traditionally categorized into type I and type II tumors. Most cases of EC are Type I, which makes up around 80% of all cases [2]. Hysterectomy is the most common form of treatment. However, once the uterus is removed, young patients will lose their fertility. In addition, some early-stage malignancies recur and spread to other organs. Surgical treatment alone is not effective for these patients, and usually requires a combination of radiotherapy and chemotherapy. The prognosis is poor, and it is necessary to establish a new treatment strategy to overcome these issues.

Hormone therapy with progestin is a non-invasive treatment. Clinically, high-dose progestin is widely used in the conservative treatment of AEH and EC to preserve fertility, as well as in patients with advanced EC [3]. It has been demonstrated that progestin treatment has a high efficacy in EH and a medium efficacy in primary endometrial adenocarcinoma, but a poor efficacy in advanced and recurring diseases [4]. The most commonly used progestins today include medroxyprogesterone acetate (MPA), megestrol acetate (MA), and levonorgestrel intrauterine system (LNG-IUS) [5]. Progestin therapy has a full remission rate of around 70%, but 30% of individuals are insensitive to progestin therapy, which is also known as progestin resistance [6]. So far, the underlying mechanisms of progestin resistance remain unclarified. This review aims to elucidate the therapeutic role of progestin in EH and EC and the mechanisms and molecular biomarkers of progestin resistance. It also summarizes the approaches that have been reported to overcome progestin resistance. 

## 2. Mechanisms of Progestin Repairing Endometrial Pre-Cancer/Cancer

**Figure 1:**
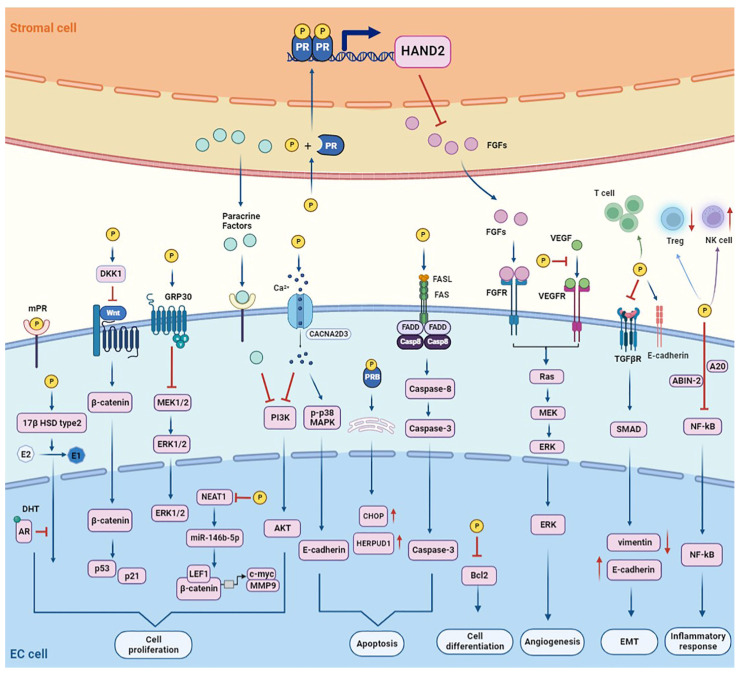
Therapeutic mechanisms of progestin in the endometrium. Progestin regulates the downstream target genes mainly by binding with PR. Progestin can also work through ER and AR. Progestin affects the cell cycle, angiogenesis, apoptosis, cell differentiation, inflammatory response, and EMT, which are all major therapeutic effects on EH and EC. Additionally, progestin can bind to PR in stromal cells to facilitate paracrine communication between stromal cells and epithelial cells.

The role of progestin in suppressing cancer growth is mostly reliant on binding with the progestin receptor (PR) and, in turn, activating the PR-mediated signaling pathways. According to the location in the cell, PR is divided into nuclear progestin receptor, membrane progestin receptor, and mitochondrial PR [7]. First, progestin binds with the inactive PR in the cytoplasm. Next, PR translocates to the nucleus, where it binds to DNA and, in turn, enhances the transcriptional activity of downstream target genes. Additionally, progestin diffuses across the nuclear membrane and binds to PR in the nucleus. Finally, PR binds to the DNA of target genes through a specific progesterone response element (PRE) and induces transcription [8]. Herein, we discuss mechanisms underlying progestin therapy in order to comprehend the contributions of progestin in treating endometrial pre-cancer/cancer (Figure 1).

### 2.1. Cell Cycle Arrest

It has been reported that progestin elicits G0/G1 cell cycle block in EC cells by regulating the lncRNA NEAT1/miR-146b-5p signal pathway [9]. The potential of progestin to suppress the expression of LEF1 and downstream genes c-myc and MMP9 in the Wnt/β-catenin signal pathway is another explanation for its inhibitory influence on G0/G1 cell cycle progression [10]. Additionally, the G2/M cell cycle block has been seen in EC cells when progestin and alsterpaullone are combined [11]. Another mechanism by which progestin inhibits the cell cycle is via binding to the G protein-coupled receptor 30 (GPR30), which directly stimulates the PI3K/AKT signaling pathway to perform non-transcriptional functions [12]. This is a non-classical pathway that rapidly activates the progestin-mediated pathway. Thus, progestin could modestly control the proliferative balance through the PI3K/AKT pathway [13].

### 2.2. Anti-Angiogenesis

It is well known that progestin exerts antiangiogenic effects in EC. Vascular endothelial growth factor (VEGF) is a glycoprotein that induces the proliferation, differentiation, and migration of vascular endothelial cells [14]. It was found that progesterone, MPA, and 17α-hydroxyprogesterone effectively inhibited the estrogen-induced production of VEGF in Ishikawa cells [15]. The Basic Fibroblast Growth Factor (bFGF) is another potent angiogenic factor. Synthetic progestins can inhibit the transcription of bFGF in the endometrial tissue [16] while antagonizing estrogen-induced production of bFGF [17]. In addition, thrombospondin-1, an anti-angiogenic glycoprotein, can be upregulated by progestin in a PR-dependent manner in Ishikawa cells [18].

### 2.3. Induction of Apoptosis

One of the essential therapeutic mechanisms of progestin treatment is the induction of apoptosis. Progestin-induced programmed cell death most likely occurs in early treatment, resulting in a decrease in the endometrial glandular epithelium [19]. Progestin stimulates the production of Fas, Fas ligand (FasL), and Fas-associated death domain (FADD), which, together, form the death-inducing signaling complex and activate the caspase-8 pathway [20]. Furthermore, progestin is thought to upregulate the expression of GPR30, resulting in the inactivation of ERK-1/2, which leads to cell death [21]. Progestin can also induce cell apoptosis by regulating ion channels. CACNA2D3, one of the Ca^2+^ channel family members, has been shown to have tumor suppressor activity in EC [22]. Progestin stimulated the CACNA2D3/Ca^2+^/p38 MAPK pathway, thus increasing the occurrence of cell death [22]. Another mechanism of progestin-induced apoptosis is the promotion of endoplasmic reticulum stress [23]. MPA may increase the expression of endoplasmic reticulum stress-related molecule HERPUD1 in Ishikawa cells by activating endoplasmic reticulum stress via the progestin-PRB pathway, then inducing cell apoptosis [24].

### 2.4. Induction of Cell Differentiation

In 1959, Kistner et al. first identified atypical secretory alteration with pseudodecidual response in the stroma in atypical hyperplasia and cancer in situ, after 3–10 weeks of progestin medication [25]. Soon after that, squamous differentiation was identified by Varga and Henriksen as a typical effect of progestin treatment [25]. Histological changes that occurred after 12 to 24 weeks of progestin therapy include various metaplasias, loss of cellular atypia, decreased glandular to stroma ratio, glandular cytopenia, and decreased mitotic activity [26]. Notably, the effect of MPA on EC cells was mainly by promoting cell differentiation and reducing cell proliferation, rather than promoting cell killing. Mechanistically, induction of EC differentiation and maturation by inhibiting Bcl-2 activity may be part of the molecular mechanism of progestin therapy. The immunoreactivity of Bcl-2 was significantly decreased after progestin medication, and tumor cell maturation was also observed in the progestin-responsive group, indicating that Bcl-2 downregulation may be closely related to squamous differentiation of EC [27].

### 2.5. Inhibition of Inflammatory Response

In the female reproductive system, progestin is a hormone with potent anti-inflammatory effects [28]. Progestin may suppress inflammation in EC, according to several studies. Treatment with progestin alters lymphocyte subpopulations in the endometrium, and may result in immunological suppression of complex atypical hyperplasia (CAH) and well-differentiated EC [29]. Progestin-treated patients had increased numbers of NK cells and decreased Tregs levels in post-treatment tissues [29]. NF-kB is a potent proinflammatory regulator that is constitutively active in cancer, and is thought to be involved in both inflammation and carcinogenesis [30]. Progestin was found to reduce NF-kB transcriptional activity in Hec50c cells by regulating the A20 and ABIN-2 proteins [31]. Additionally, progestin prevents the release of metalloproteinase, a substance known to have inflammatory properties and to be present in EC [32].

### 2.6. Inhibition of Epithelial-to-Mesenchymal Transition

Epithelium-mesenchymal transition (EMT) is a biological process in which epithelial cells lose their epithelial characteristics and obtain mesenchymal cell traits [33]. By increasing T-cell infiltration, progestin suppresses the process of EMT and metastatic spread in EC [34]. The basis of EMT includes the activation of key signaling pathways, such as Wnt/β-catenin and TGF-β. In Ishikawa cells, progestin administration suppressed TGF-β signaling and significantly reduced the survival and invasion of EC cells with elevated E-cadherin expression [35]. Bokhari et al. demonstrated that progestin reduced TGF-β-induced R-SMAD expression by decreasing vimentin expression and increasing E-cadherin expression, thereby reducing the proliferation and invasiveness of EC cells [36].

### 2.7. Regulation of Estrogen/Androgen Receptor

The physiological role of progestin in the endometrium is thought to antagonize estrogen-induced endometrial proliferation and induce cell differentiation [37]. Progestin decreases ER expression primarily by increasing ER breakdown and decreasing ER synthesis [38]. By reducing the expression of the estrogen receptor (ER) and repressing ER-related transcription of genes involved in cell growth, progestin inhibits the proliferation of EC cells [39]. Furthermore, the 17β-hydroxysteroid dehydrogenases (17-HSDs) are enzymes that play a role in the synthesis of androgens and estrogens [40]. Progestin may also suppress the proliferation of endometrium by promoting the induction of 17-HSD type 2, which converts effective estrogen E2 to ineffective estrogen E1 and PR, especially PRB [41]. Therefore, in situ abundance of 17-HSD type 2 can be used to predict the response of endometrium to progestin [42]. 

Furthermore, many investigations have reported the androgen-agonistic effects of MPA on mammalian cells expressing exogenous or endogenous androgen receptor (AR). AR overexpression and MPA treatment led to markedly elevated AR transcriptional activity in the steroid receptor-negative COS-1 cell line [43]. Furthermore, progestin may inhibit the stimulation of estrogen signaling by upregulating the expression of AR in the endometrium [44]. It has been reported that MFE-296 EC cells express AR in vitro, and both progestin and dihydrotestosterone (DHT) treatment can inhibit the proliferation of MFE-296 cells [45].

### 2.8. Progestin Induced Paracrine Regulation

The role of progesterone in EC is mainly focused on cancer cells, but little is known about the role of the stroma. In the endometrium, circulating progestin first comes into contact with multilayered stromal cells that contain PR [46]. A significant decrease in hormone-stimulated PI3K/AKT signaling in Ishikawa cells was induced by paracrine factors from normal endometrial stromal cells [47]. Another study found that the coculture of stromal cells with Ishikawa cells increased cell differentiation and glycodelin expression, independent of hormone treatment [48]. Furthermore, the progestin-mediated paracrine mechanism is found to suppress estrogen-induced uterine epithelial DNA synthesis [49]. Endometrial stromal cells treated with progestin secrete paracrine factors that enhance the levels of 17-HSD type 2, while direct progestin treatment did not affect the enzyme activity of Ishikawa cells [41]. HAND2, as a PR-regulated gene, is expressed in the uterine stroma. HAND2 suppresses the expression of fibroblast growth factor (FGF) or inhibits the response of FGF-10 in the stromal to its receptors to counteract estrogen-induced endometrial proliferation [50,51].

## 3. Mechanisms of Progestin Resistance in Endometrial Pre-Cancer/Cancer

**Figure 2:**
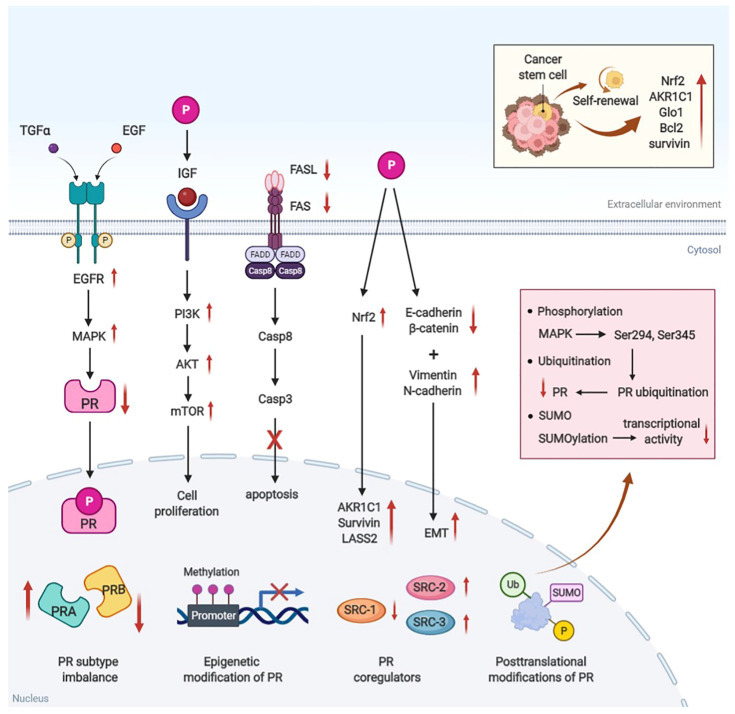
Mechanism of progestin resistance related to PR signal pathway. The mechanisms of PR signaling mainly include the following categories: PR subtype imbalance, epigenetic modification of PR, aberrant expression of PR coregulators, posttranslational modifications of PR, and abnormal PR-associated classic pathways. The traditional pathways connected to PR include the TGF/EGFR/MAPK pathway, the PI3K/AKT/mTOR pathway, the tumor apoptosis pathway, the Nrf2 pathway, and the aberrant EMT pathway.

**Figure 3:**
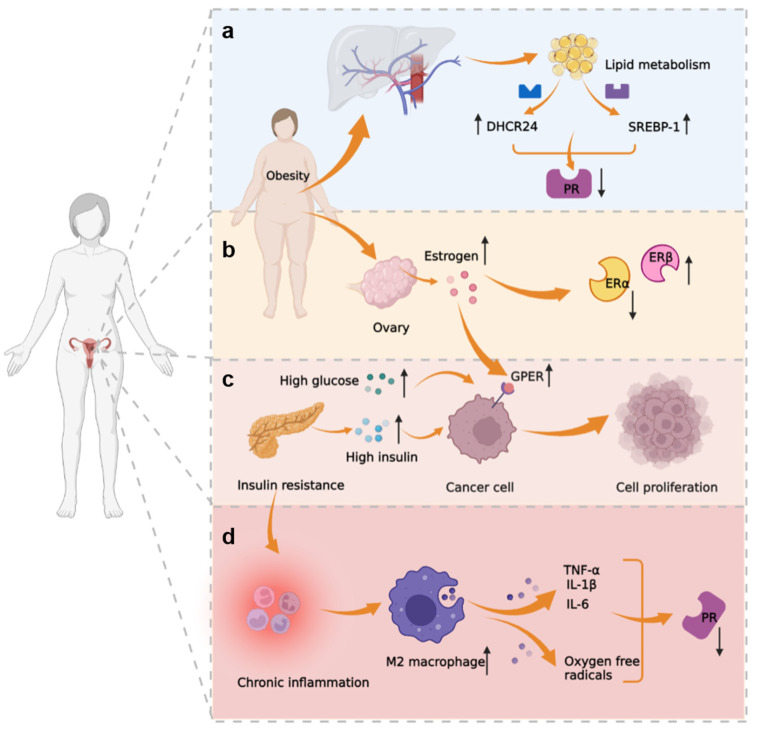
Mechanism of progestin resistance related to metabolic-immune-tumor microenvironment. (**a**) Lipid metabolism-related enzymes SREBP-1 and DHCR24 regulate PR expression. (**b**) Obesity promotes estrogen production in ovaries. In progestin-resistant EC cells, ERα expression is downregulated and ERβ expression is increased. (**c**) Obesity, insulin resistance, and high glucose levels can upregulate GPER and make tumor cells more estrogen-sensitive. (**d**) Infiltration of M2 macrophages is more common in EC, and through secreting TNF-α, IL-1β, IL-6, and oxygen free radicals, they can suppress PR expression.

Although a large number of studies have shown that progestogens can be used for conservative treatment of AEH and EC with good therapeutic effects, progestin resistance still affects a large number of individuals. Progesterone resistance presents significant challenges to conservative management. We compiled recent research on progestin resistance in EH and EC to gain a deeper understanding of the mechanisms involved. Aberrant PR signaling (Figure 2), other abnormal signaling pathways, metabolic-immune-tumor microenvironment (Figure 3), and EC stem cells are the major causes of progestin resistance in endometrial pre-cancer or cancer.

### 3.1. Aberrant PR Signaling

*Decreased PR expression and subtype imbalance.* The expression of PR is positively correlated with the response to progestin therapy and the prognosis of patients with EC. In patients with high PR expression, the overall response rate of progestin therapy was 72%, while the response rate in patients with PR-deficient tumors was only 12% [52]. PRA and PRB are two structurally similar subtypes. PRB may be the main isoform responsible for the tumor-suppressing effects of progestin. MPA treatment inhibited proliferation, migration, and invasion in both Ishikawa and Hec50co cells stably transfected with PRB, but not in these same two EC cells stably transfected with PRA [53]. High PRB expression in AEH patients is associated with a lower probability of progestin resistance [54]. Additionally, the researchers discovered that women who reacted to MPA had higher levels of PRA and PRB expression, or a higher ratio of PRB/(PRB+PRA) (Figure 2) [42]. Therefore, reduced PR expression, especially PRB expression, can be used to predict progestin-resistant tumor subsets.

*Epigenetic modification of PR.* CpG islands are located in the 5’ upstream region of the PR promoter, and abnormal methylation of the CpG island silences the expression of related genes [55]. Aberrant methylation of DNA promoters and exons reduces PR expression in EC, inhibits PR production at the transcriptional level, further reduces progestin sensitivity, and leads to poor prognosis (Figure 2) [55]. Even though both PRA and PRB are produced by transcription of the PR gene, different promoters control PRA and PRB, which may lead to variations in the epistatic modification patterns of the two receptor isoforms. Increasing evidence has shown that PR methylation alterations are most evident in the PRB promoter region, and are associated with decreased PRB production and reduced progestin sensitivity in EC [56].

*Post-translational modifications (PTMs) of the PR.* Common forms of PTMs include phosphorylation modifications, ubiquitination modifications, and SUMO-like modifications (Figure 2) [57]. PR stability, subcellular location, transcriptional activity, and target genes can all be impacted by PTMs. There are several serine (Ser) phosphorylation sites at the amino terminus of PR, which can be selectively phosphorylated by a variety of kinases. For instance, the Ser294 and Ser345 sites of PRB are specifically phosphorylated by MAPK, which promotes the nucleation of the PR and activates its control of target genes [57]. Meanwhile, Ser294 phosphorylation leads to changes in PR ubiquitination, resulting in ligand-dependent PR protein degradation [58]. Although ubiquitination can lead to PR downregulation, studies have revealed that ubiquitination degradation is also necessary for PR activation through downstream signaling pathways. Yang et al. reported that the MAPK inhibitor PD0325901 inhibited PR phosphorylation in EC cells and reduced ubiquitination-mediated PR degradation [59]. However, undegraded PR has no transcriptional activation effect. As a result, PR positivity does not imply progestin sensitivity. Another key PTM of the steroid receptor is SUMOylation. There is a sumo-binding consensus sequence at the k388 site at the amino terminus of PR, and SUMOylation inhibits transcriptional activity by 6–10-fold compared to the non-SUMOylated PR [60].

*Abnormal expression of PR coregulators.* The recruitment of co-regulatory factors, which may be divided into transcription-promoting co-stimulators and transcription-inhibiting co-repressors, is necessary for steroid receptors to control their target genes [61]. The co-regulatory factor complex has chromatin-remodeling enzyme activity and can produce local acetylation or deacetylation of chromatin. Co-regulatory factors operate as a “bridge” between PR and chromatin DNA. SRC-1, SRC-2, and SRC-3 are essential coregulators that regulate PR transcriptional activity and response to progestin in EC patients (Figure 2) [61]. Several studies have shown that PR co-regulators are abnormally expressed in EC, manifesting with abnormally high expression of SRC-2 and SRC-3, low expression of SRC-1, and reduced expression of PR, and thus resulting in poor progestin responsiveness [62]. Therefore, the decreased progestin responsiveness of tumor cells may be mediated by aberrant expression of PR co-regulators in the EC microenvironment. The specific molecular mechanisms are unknown, but they may be related to mutations in the functional area of PR’s first activation function (AF1) and decreased binding of co-regulators [63].

### 3.2. Other Abnormal Signaling Pathways

*TGF/EGFR/MAPK pathway.* Increased epidermal growth factor receptor (EGFR) expression results in decreased PR expression, decreased sensitivity of Ishikawa cells to progestin, and inappropriate activation of the MAPK signaling pathway (Figure 2) [64]. Ai et al. discovered that progestin resistance in EC is associated with an increase in endogenous growth factors such as TGFɑ/EGF, EGFR, and EGFR tyrosine kinase (EGFR-TK) [64]. TGFα can trigger EGFR autophosphorylation, which increases EGFR-TK activity, activates the intracellular MAPK signaling pathway, decreases PR expression, and ultimately leads to cell division and proliferation. Another study discovered that EGFR levels in PRB-negative EC specimens were higher than in PRB-positive cases [65]. These results imply that the decrease in progestin sensitivity in EC cells is associated with EGFR overexpression.

*PI3K/AKT/mTOR pathway.* Activation of the PI3K/AKT pathway by progestin can also lead to progestin resistance of EC cells, and blocking this pathway is considered to be a potential strategy to overcome resistance. The PI3K/AKT/mTOR pathway is associated with autophagy. FAM83B inhibits autophagy via activating the PI3K/AKT/mTOR pathway to promote EC cell proliferation and metastasis [66]. In progestin-sensitive cells, MPA blocked the PI3K/AKT pathway and inhibited cell proliferation, whereas in progestin-resistant cells, MPA activated this pathway through a PR-independent route without inhibiting cell proliferation (Figure 2) [67]. Furthermore, Dong et al. discovered that FKBP51 overexpression reduced cell proliferation and enhanced progestin sensitivity by inhibiting the AKT signaling pathway in EC [68]. Therefore, the PI3K/AKT/mTOR pathway is one of the mechanisms of progestin resistance in EC [69].

*Tumor apoptosis pathway.* Apoptotic-related pathways appear to be inhibited when EH or EC is progestin-resistant. Fas-FasL interaction is one of the key mechanisms in the regulation of cell apoptosis. According to some studies, progestin inhibits the growth of cancer cells by increasing the expression of Fas and FasL in the endometrial glandular epithelium [70]. In EC patients who were not sensitive to progestin, the expression of Fas and FasL was downregulated, which may be a factor in EC progestin resistance (Figure 2) [70]. Furthermore, after therapy, the expression of Bcl-2 was dramatically reduced in individuals whose EH returned to normal after treatment with progestin. However, Bcl-2 expression in women with persistent EH after progestin treatment did not change significantly before or after treatment [71].

*Nrf2 and oxidative stress pathway.* Nrf2 is a vital nuclear transcription factor that regulates the transcription of many target genes to maintain intracellular redox equilibrium [72]. Nrf2 plays a significant role in chemotherapeutic resistance in a wide variety of cancers [73]. A high level of Nrf2 has also been discovered in endometrial carcinoma, which has led to progestin resistance by regulating downstream target genes (Figure 2) [74]. Furthermore, oxidative stress mediates progestin resistance through the regulation of PR. It is reported that the generation of free radicals and the activation of oxidative stress signal pathways can affect the functional state of the receptor by post-translational alterations of the PR [75].

*Epithelial-to-mesenchymal transition pathway.* EMT is the process through which cells acquire the migratory and invasive qualities present in low-differentiated stem cells that proliferate quickly. The so-called “Cadherin switch,” which is characterized by decreased E-cadherin and increased N-cadherin, is the distinguishing feature of EMT [76]. Microarray analysis between Ishikawa and progestin-resistant cells (Ishikawa-PR cells) revealed that the mesenchymal markers Vimentin and N-cadherin were elevated in Ishikawa-PR cells, while epithelial markers E-cadherin and β-catenin were significantly reduced (Figure 2) [77]. This is the first time that EMT has been linked to acquired progestin resistance in EC.

### 3.3. Metabolic-Immune-Tumor Microenvironment

*Disorder estrogen-related signal pathway.* Currently, two ER isoforms have been identified: ERα and ERβ. In tumors xenografted from the Ishikawa cells, high levels of ERα dramatically suppressed tumor development and decreased VEGF expression [78]. Progestin-resistant Ishikawa cells had a lower positive expression rate of ERα and PRB, and a higher positive expression rate of Erβ, when compared to Ishikawa cells (Figure 3) [79]. Therefore, an imbalance of ER subtypes may play a role in the progestin resistance mechanisms. Excessive peripheral and local estrogen production also leads to progestin resistance. Studies have demonstrated that obesity decreases the expression of sex hormone-binding globulin (SHBG), which results in higher levels of estradiol in the blood. Obesity also increases the synthesis of estrogen in the ovary and surrounding adipose tissue [80]. Furthermore, high glucose stimulates the EMT process and accelerates the development of EC by upregulating ER [81]. A high insulin microenvironment can upregulate the expression of G-protein-coupled estrogen receptor (GPER) in EC cells, increasing the sensitivity of tumor cells to estrogen and facilitating tumor cell proliferation (Figure 3) [82].

*Chronic inflammation of endometrium.* In recent years, chronic inflammation and the role of cytokines have become increasingly important in the etiology of EC. Researchers found that the A disintegrin and metalloproteinase with thrombospondin motifs (ADAMTS) family can interact with inflammatory cytokines. The expression of ADAMTS5 was significantly increased in patients with EC, and ADAMTS5 led to extracellular matrix degradation [83]. The relationship between progestin resistance, metabolism, and immunity was first confirmed by Li et al. when they used microarray analysis technology to screen the differential genes of progestin-resistant and progestin-sensitive EC cells. They discovered that those differential genes were primarily enriched in fat metabolism, immune function regulation, and inflammation-related pathways [84]. Obesity and insulin resistance can cause a chronic inflammatory state in the body, and have an impact on the immunological function of immune cells such as macrophages, CD8+T cells, and NK cells in the tumor microenvironment. M2 macrophages infiltrated EC more significantly than in a normal proliferative endometrium. Multiple cytokines, including TNF-α, IL-1, IL-6, and oxygen-free radicals, which are released by M2 macrophages, promote the development of EC (Figure 3). Through epigenetic changes, TNF-α and IL-1β may directly suppress PR expression. Pro-inflammatory substances may potentially compete with PR as co-regulators and alter the regulatory function of progestin [85].

*Endocrine metabolic disorder.* The occurrence and development of EC are closely related to abnormal metabolism. Recently, some scholars have confirmed that AEH patients with insulin resistance and overweight require longer progestin treatment, indicating that metabolic disorders can inhibit the body’s response to progestin (Figure 3) [85]. Aromatase, which is upregulated in obese women, converts androstenedione into more estrogen, thus requiring more progestin to counteract the effects of estrogen [86]. In addition, BMI affects the complete response time to conservative treatment. Park et al. believed that BMI ≥ 25 kg/m^2^ was significantly related to the low complete response rate of progestin treatment and the high recurrence rate after treatment [87]. Lipid metabolism may also be related to the mechanism of progestin resistance. SREBP-1 is a rate-limiting enzyme that regulates cholesterol and fatty acid synthesis. SREBP-1 was upregulated in progestin-resistant cells, while PR was downregulated (Figure 3) [88]. Insulin resistance is an important metabolic-related mechanism leading to progestin resistance [89]. It was found that both insulin-like growth factors (IGF)-I and IGF-II inhibit PRA/B, whereas metformin markedly promotes PR expression in patients with AEH and EC [90]. In addition, insulin can mediate progestin resistance by affecting enzymes of lipid metabolism. DHCR24, the final enzyme of the cholesterol metabolism pathway, was significantly increased in EC patients, correlated with decreased clinical stage and overall survival, and negatively correlated with PR expression in cells (Figure 3) [91]. Insulin can induce DHCR24 expression by STAT3, therefore promoting EC cell metastasis and progestin resistance [91]. 

### 3.4. Endometrial Cancer Stem Cells

Endometrial cancer stem cells (CSCs) are distinguished by their ability to self-renew and differentiate into mature cells of a specific tissue [92]. Several studies have reported that the presence of CSCs in target tissue may be a significant additional factor causing various treatment resistances [93]. Dysregulation of stem cell function may play a role in the etiology of endometrial proliferative disorders [94]. When Ishikawa cancer stem cells were treated with different dosages of MPA, no growth inhibition was found, and only a small reduction in cells was observed in ECC1-CSCs [95]. Furthermore, the expression of progestin resistance-associated markers, such as AKR1C1, Nrf2, Glo1, Bcl2, and survivin were shown to be higher in CSCs than in parental EC cells (Figure 2) [95]. These findings suggest that CSCs are an important target for overcoming progestin resistance.

## 4. The Molecular Biomarkers of Progestin Resistance

**Figure 4:**
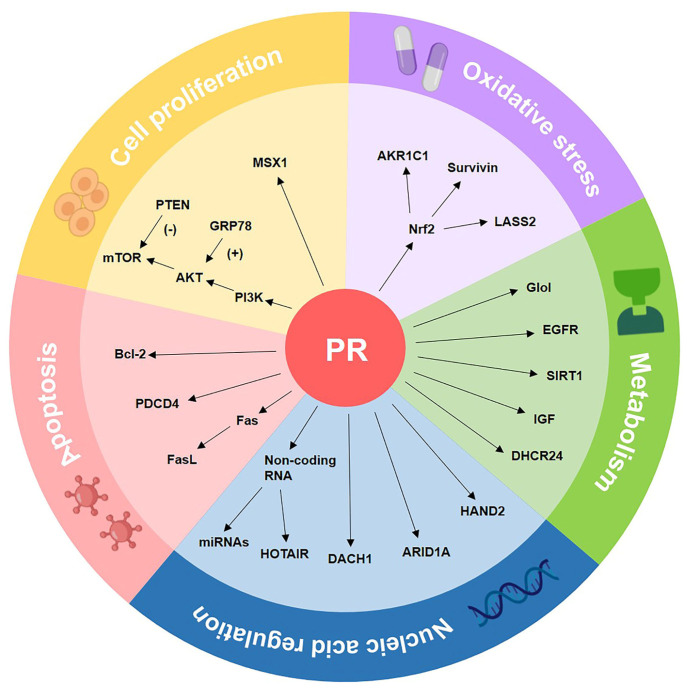
The molecular biomarkers of progestin resistance. Numerous biomarkers may be categorized into five basic groups: molecules linked to cell proliferation, molecules related to oxidative stress, molecules related to metabolism, molecules related to the apoptosis pathway, and nucleic acid regulation-related biomarkers.

Patients’ responses to progestin therapy cannot be accurately predicted due to progestin resistance. Several clinical factors can be used to predict treatment response to oral progestins, such as obesity, polycystic ovary syndrome, history of infertility, and longer menstrual cycles [96,97,98]. Significant research efforts, including our own, have been conducted to more effectively select suitable candidates who would respond favorably to progestin treatment. We summarized these markers, reported over the years, as listed in Table 1, and divided them into the following categories according to biological functions: cell proliferation, oxidative stress, metabolism, apoptosis, non-coding RNA, and nucleic acid regulation (Figure 4).

### 4.1. Cell Proliferation-Associated Biomarkers

*PI3K/AKT/mTOR.* The PI3K/AKT/mTOR pathway is essential for the growth of EC and other tumor types [99]. Gain-of-function mutations of PI3KCA cause the PI3K/AKT pathway to be constitutively activated, which, in turn, causes mTOR to become hyperactivated [99]. Mutations of the PIK3CA gene are found in 2% to 14% of type I endometrial cancers, and increased signaling of the PI3K/AKT/mTOR pathway is associated with a poor prognosis in both type I and type II carcinomas [100]. Liu et al. suggested that long-term progestin therapy may result in progestin resistance via activating the PI3K/AKT/mTOR signal pathway [101]. Blocking the PI3K/AKT/mTOR pathway promotes autophagy and makes EC cells more responsive to progestin. Additionally, it has been suggested that mTOR activation promotes progestin resistance. Suppressing the mTOR pathway can inhibit tumor growth by reducing cell proliferation and inducing apoptosis and autophagy, eventually reversing progestin resistance [102].

*PTEN.* PTEN is a tumor suppressor protein that dephosphorylates the PI3K/AKT/ mTOR pathway, resulting in lower downstream activation of mTOR [103]. Mutations in PTEN have been found in 34–83% of human ECs and up to 55% of EH [104]. Milam et al. discovered that after progestin therapy, persistent hyperplasia unresponsive to progestin medication was related to both PTEN loss and mTOR phosphorylation [105]. PTEN deficiency activates AKT, which phosphorylates the downstream protein mTOR, resulting in cell proliferation [106]. Therefore, PTEN deficiency is a good predictor of progestin responsiveness.

*GRP78.* GRP78 belongs to the heat shock protein-70 (HSP70) family, and is an important endoplasmic reticulum chaperone protein [107]. It is found that GRP78 overexpression is more common in endometrioid cancer than in normal endometrium within the uterus [108]. In CAH samples, high GRP78 expression indicated a poor response to progestin treatment [108]. Furthermore, GRP78 is an upstream regulator of PI3K/AKT signaling. Overexpression of GRP78 promoted the activation of AKT and further regulates the PI3K/AKT signaling pathway, which may be one of the reasons for the suboptimal response to progestin in EC [109].

*MSX1.* MSX1 has been reported to be a transcriptional repressor that regulates the cell cycle, and is thought to play an important role in the development of EC [110]. Endometrial neoplasms have the greatest expression of MSX1, demonstrating the strong tissue specificity of this gene [111]. In vitro research by Yang et al. revealed that MSX1 was substantially more elevated than other candidates in progestin-resistant Ishikawa-PR cells, and its knockdown improved the efficacy of progestin therapy [111]. The mechanism by which MSX1 contributes to progestin resistance has not been elucidated. MSX1 may affect EC progression through the p53 pathway. In conclusion, the MSX1 gene was expected to be a precise progestin resistance biomarker and therapeutic target.

### 4.2. Oxidative Stress-Related Biomarkers

*Nrf2.* Nrf2 is known as a key regulator of cell response to oxidative stress, and binds to the antioxidant response element to activate genes that are crucial for protecting cells from oxidative stress [112]. Nrf2 has been shown to contribute to progestin resistance of EC [113]. Yang et al. discovered that EC cells would become more responsive to progestin therapy if Nrf2/LASS2 expression were reduced [114]. The Nrf2-survivin pathway is also crucial for progestin resistance in individuals with endometrial precancers or malignancies [115]. It was found that progestin resistance was caused by exogenous overexpression of Nrf2 and survivin. Furthermore, as a particular inhibitor of Nrf2, brusatol was able to reverse progestin resistance in EC cell lines with a decrease in Nrf2/AKR1C1 [116]. In conclusion, Nrf2 is likely one of the primary markers of progestin resistance in patients with EH and EC.

*AKR1C1.* AKR1C1 is one of the four human AKR1C isoenzymes whose main physiological function is to convert progesterone to its inactive form, 20α-dihydroxyprogesterone [117]. Overexpression of AKR1C1 may cause increased progestin catabolism, which may weaken progestin signaling by its nuclear receptors [118]. AKR1C1 binds to the promoter of PRB, resulting in reduced progesterone-dependent PR activation [118]. In addition, as one of the Nrf2 target genes, AKR1C1 mediates Nrf2-driven progestin resistance. Nrf2 and AKR1C1 were exclusively overexpressed in EC samples that were not responding or partially responding after progestin treatment, but Nrf2 and AKR1C1 expression was absent in the endometrial samples that had a full response [116]. 

*Survivin.* Survivin is an inhibitor of apoptosis proteins with multiple functions, such as regulation of cell division, cell death, and angiogenesis [115]. Survivin is expressed in both normal and proliferative human endometrium, and it is overexpressed in hyperplastic and malignant endometrium. In EC cells, exogenous survivin overexpression caused progestin resistance [115]. Additionally, one study indicated that patients with EH were more likely to develop resistance to progestin medication if survivin protein levels were higher [119]. Therefore, targeting survivin may represent a promising prevention and treatment strategy for EH and EC. 

*LASS2.* LASS2, a ceramides synthesizer with widespread tissue distribution, is engaged in multiple intracellular signaling processes such as apoptosis, senescence, proliferation, growth, and differentiation [120]. LASS2 is also one of the downstream target genes of Nrf2, and is involved in the regulation of progestin resistance. Overexpression of Nrf2/LASS2 resulted in progestin resistance in EC [114]. Knockdown of LASS2 increased tumor cell apoptosis and decreased cell survival, resulting in a more effective progestin treatment. Furthermore, metformin overcomes progestin resistance by downregulating Nrf2/LASS2 expression. Thus, overexpression of LASS2 may serve as a valuable biomarker to estimate the potential progestin resistance of EC cells. 

### 4.3. Metabolism-Related Biomarkers

*GloI.* Glyoxalase I (GloI) is a part of the glyoxalase system, and it is reported to result in progestin resistance in EC [121]. Elevated GloI expression is associated with cancer cell proliferation and chemoresistance [122]. Silence of GloI improved MPA’s ability to suppress cell proliferation in progestin-resistant Ishikawa cells. In EC cells, GloI is also a target gene of metformin, and downregulated GloI is associated with metformin’s ability to reverse progestin resistance [121]. In addition, siRNA-mediated knockdown of TET1 reduced the expression of GloI, suggesting that GloI is a target gene of TET1. As a result, metformin increased progestin sensitivity in EC via the TET1-GloI signaling pathway [123].

*EGFR.* Epidermal growth factor receptor (EGFR) is an important member of the ErbB/ HER receptor tyrosine kinase family, and is involved in the development of cancer [124]. Evidence showed that progestin-resistant EC cells express less PRB than non-progestin-resistant EC cells, whereas progestin-resistant EC cells express more EGFR than non-progestin-resistant EC cells [125]. By triggering the downstream PI3K/AKT signal transduction, the elevated EGFR can promote the proliferation and metastasis of malignancies [126]. Moreover, Studies indicate that the EGFR is overexpressed in the EC cells with progestin resistance, and the upregulated EGFR contributes to the downregulation of PRB, resulting in progestin resistance in EC patients [127].

*SIRT1.* Sirtuin 1 (SIRT1) is a nicotinamide adenine dinucleotide (NAD)-dependent deacetylase which deacetylates the lysine residues of multiple histones and non-histone proteins [128]. Sirtuins are widely distributed throughout the cell, and are involved in cell proliferation, inflammation, and metabolism, among other processes [129]. SIRT1 plays a dual role of tumor suppressor or tumor promoter in different tumors [130]. It has been demonstrated that SIRT1 is overexpressed in EC compared with normal endometrium [131]. Moreover, in progestin-resistant cells, SIRT1 was upregulated, whereas PR and FoxO1 were downregulated; therefore, SIRT1 knockdown cells were shown to be more sensitive to MPA than progestin-resistant cells [88].

*DHCR24.* DHCR24 is the final enzyme of the cholesterol biosynthesis pathway, which catalyzes the reduction of the Δ 24 double bond in desmosterol to generate cholesterol [132]. DHCR24 regulates multiple cellular functions, such as anti-apoptosis, oxidative stress, and cell differentiation [133]. Dai et al. found that DHCR24 expression levels were significantly elevated in EC patients, and that upregulated DHCR24 was associated with advanced EC, lymph node metastasis, and decreased overall survival [91]. DHCR24 overexpression displayed progestin resistance traits, and DHCR24 silencing could significantly upregulate the expression of PR, making EC cells more susceptible to MPA [91]. Therefore, DHCR24 could be targeted in order to develop pharmacological strategies to enforce responsiveness to progestin in EC patients.

*IGF.* Accumulating evidence suggests that diabetes and insulin resistance are oncogenic factors for EC. The insulin-like growth factor (IGF) system plays a crucial role in the initiation and progression of EC. It was discovered that EC had substantially greater levels of IGF-II and IGF-I receptor (IGF-IR) than did normal endometrium [134]. IGF-IR binds to the ligands IGF-I, IGF-II, or insulin, induces autophosphorylation, and activates downstream signaling pathways, including the PI3K/AKT/mTOR pathway [135]. IGF-II enhances the phosphorylation of AKT and p70S6K, and significantly promotes cell proliferation in EC [90]. Importantly, both IGF-I and IGF-II downregulate the mRNA and protein levels of PR, resulting in EC being insensitive to progestin therapy [90].

### 4.4. Apoptosis Pathway-Related Biomarkers

*Fas/FasL.* Fas is a type I membrane protein, and the interaction of Fas with FasL is one of the important events in the induction of apoptosis [136]. Endometrial cycling depends on Fas-mediated apoptosis, which suggests that deregulation of the Fas/FasL interactions may play a significant role in the emergence of EC [137]. Depressed cellular responsiveness to progestin effects may be caused by imbalanced Fas/FasL system expression in hyperplastic endometrium [70]. Furthermore, Fas/FasL dysregulation may potentially contribute to the formation of progestin-resistant cells, and elevated tissue levels of Fas expression may be used as a measure of the effectiveness of the progestin therapy [70].

*Bcl-2.* Bcl-2 is an oncogene that prolongs cell viability by inhibiting cell apoptosis [138]. It is reported that Bcl-2 contributes to endometrial homeostasis by regulating apoptosis in the endometrium [71]. In a study of low-grade EC treated with progestin, Bcl-2 was downregulated, concomitant with tumor cell maturation and reduced Ki-67 staining [27]. The expression of Bcl-2 declines after effective progestin treatment of EH, whereas it remains expressed in hyperplasia, which persisted despite progestin therapy [71]. In addition, after oral administration of progestin, the expression of Bcl-2 in stromal cells appears to be a possible biomarker that can distinguish between progestin therapy responders and non-responders [139].

*PDCD4.* PDCD4 was first known as an apoptosis-related gene, and is now identified as a tumor suppressor gene that inhibits neoplastic transformation and tumor development [140,141]. It has been reported that PDCD4 is a target gene of progestin therapy, and its expression can reflect whether patients with EC are sensitive to progesterone therapy [142]. Progestin inhibits the production of the PDCD4 protein through the PI3K/AKT signaling pathway in EC [142]. Given that PDCD4 is a specific type of tumor suppressor, downregulation of PDCD4 by progestin may be one of the factors contributing to progestin’s poor therapeutic effectiveness and association with progestin resistance [142].

### 4.5. Nucleic Acid Regulation-Related Biomarkers

*MicroRNA.* A microRNA is a short RNA molecule, 21–25 nucleotides in length, that does not encode a protein [143]. Progestin has been reported to interact with miRNAs. Some miRNAs affect progestin production and regulate PR expression, while others are regulated by progestin [144]. The efficiency of hormone treatment in EC cells is reported to be constrained by five miRNAs (miR-96, miR-182, miR-141, miR-129-5p, and miR-375), which adversely correlate with PR expression in endometrial tissues [59]. miR-96 has the most significant effect on PR. Transient transfection of anti-miR-96 into Ishikawa cells revealed increased mRNA expression of PR as well as increased expression of PR downstream target genes [59].

*LncRNA.* HOX transcript antisense intergenic RNA (HOTAIR) is one of the well-known lncRNAs, with a length of 2158 bp [145]. HOTAIR and PRB expression were negatively linked, and HOTAIR mediated progestin sensitivity by suppressing PRB expression in EC [146]. Furthermore, in EC cells treated with MPA, HOTAIR knockdown increased PRB transcription by recruiting LSD1 to the PRB promoter, which resulted in H3K4me2 demethylation at the PRB promoter and the suppression of proliferation. In conclusion, HOTAIR is a possible predictor of progestin responsiveness in EC [146].

*DACH1.* DACH1 is a highly conserved nuclear protein that regulates hormone receptor signaling in a variety of hormone-responsive cancers, such as breast cancer and prostate cancer [147,148]. An earlier study found that DACH1 is less expressed in EC than in normal endometrium, suggesting that DACH1 may play a tumor-suppressing role in EC [149]. According to Zhou et al., the expression of DACH1 was positively correlated with PR, and the knockdown of DACH increased proliferative potential, metastatic capacity, and progestin resistance in Ishikawa cells [77]. On the other hand, DACH1-overexpressing Ishikawa-PR cells became more sensitive to progestin therapy. 

*ARID1A.* ARID1A, as one of the members of the SWI/SNF chromatin remodeling family, is frequently mutated in EH and EC (26–40%) [150]. Up to 16% of CAH and 40% of EC have an ARID1A mutation, and women who are diagnosed with EC at a younger age tend to have these mutations more frequently [151]. By excessively stimulating the PI3K/AKT signal pathway and downregulating the expression of PRB in EC, ARID1A knockout induced progestin resistance [152]. Furthermore, it is reported that ARID1A deficiency contributes to the loss of PR in late-stage EC, which leads to progestin resistance and an elevated risk of EC [153]. 

*HAND2.* HAND2 is a potential PR-regulated gene that is activated by progestin and inhibits estrogen-mediated endometrial epithelial growth, thereby exerting a tumor suppressor role in EC [50]. Additionally, the expression of HAND2 is strongly suppressed in EC by DNA methylation [51]. HAND2 methylation levels were significantly higher in patients who did not respond to progestin therapy compared to those who responded to progestin therapy [51]. Therefore, HAND2 methylation, a common and significant molecular change in EC, can serve as a biomarker for early diagnosis and a predictor of treatment response.

### 4.6. Biomarkers of Endometrial Cancer with Different Molecular Types

In 2013, The Cancer Genome Atlas (TCGA) classified EC into four categories: POLE ultramutated, microsatellite instability (MSI) hypermutated, copy-number low, and copy-number high [154]. Type I tumors comprise the “low copy number” TCGA category, which typically has PTEN mutations, and are associated with better differentiation and higher PR expression levels. Type II tumors, on the other hand, comprise the “high copy number” TCGA category, and are characterized by p53 mutations and PR loss [155].

Molecular typing makes it possible for doctors to make treatment decisions earlier and more precisely. According to research, the effect of treatment in patients with p53 wild-type and pole mutant is better, which may be due to the increased PR expression [156]. The majority of patients with p53 mutation are not sensitive to progestin therapy. Lynch syndrome patients usually have higher progestin resistance [156]. According to a cohort study, progestin resistance of EC in patients with Lynch syndrome was related to abnormal expression of mismatch repair (MMR) protein, and progestin therapy did not affect the expression of MMR in endometrial tissue. Additionally, the study discovered that the tumor regression rate of patients with normal MMR expression was significantly higher than that of patients with abnormal MMR expression [157]. MMR deficiency is responsible for MSI [158]. MMR seems to be a progestin-resistance marker of MSI. Previous research revealed that more than 90% of patients with MMR-deficient AEH and endometrioid endometrial cancer were resistant to conservative therapy [157,159]. 

## 5. Potential Therapeutic Methods to Enhance Progestin Sensitivity

**Figure 5:**
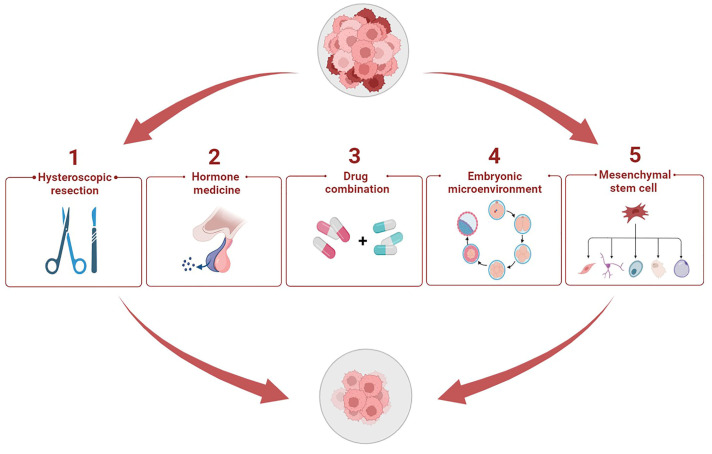
Potential therapeutic methods to reverse progestin resistance. Progestin resistance can be improved in a number of ways. There are five main types of treatment, namely hysteroscopic resection, hormone medicine, drug combinations, cytokines in the embryonic microenvironment, and mesenchymal stem cells.

The therapeutic effect of progestins is limited, and long-term administration of progestins can lead to failure of conservative treatment. Therefore, clinicians are exploring new treatment methods to improve progestin resistance (Figure 5). Notably, in this review, we list many registered clinical trials on progestin therapy in recent years (Table 2), which can provide new ideas for future research directions of the conservative treatment of EH and EC.

### 5.1. Hysteroscopic Resection plus Progestin

Hysteroscopic endometrial lesion excision allows for the removal of the lesion as well as the accurate assessment of the location and extent of the endometrial lesion, in order to achieve a definitive diagnosis. Hysteroscopic resection combined with progestin is safe and effective in the treatment of young patients with early EAH and EC. Approximately 90% of patients respond completely to treatment, and approximately 33% of patients have a successful pregnancy [160]. Consistent evidence has shown that the combination of hysterectomy and progestin therapy has better efficacy than progestin alone [161]. In addition, when hysterectomy was combined with LNG-IUS, at least similar remission and live birth rates were obtained as with progestin alone, while recurrence rates were significantly lower [162].

### 5.2. Hormone Medicine

*Tamoxifen.* Tamoxifen is a selective estrogen receptor modulator that induces PR expression. Tamoxifen combined with progestin can enhance the therapeutic effect of progestin on EC [163]. In an athymic mouse model with subcutaneous human endometrial adenocarcinoma, tamoxifen showed the same ability to regenerate PR as estradiol, but tamoxifen significantly reduced the growth of tumor cells [163]. Moreover, the Gynecologic Oncology Group reported the strategy of combining tamoxifen with intermittent progestin treatment, and the response rates with this approach reached 33% in cases of advanced EC [164]. Unfortunately, the tumors eventually became resistant to hormonal therapy in both the mouse model and in patients [165].

*Mifepristone (MF).* MF is an anti-progestin with a two- to ten-fold higher affinity for PR than progestin, but it also acts as a PR agonist [166], thereby enhancing the antitumor effects of MPA [167]. This could account for the antiproliferative effects of MF on the human EC cell line RL95-2 and the benign endometrial cell line EM42 [168,169]. More importantly, the combination of MF and MPA was more effective in inhibiting the proliferation of EC than MF or MPA alone [170]. Another study has shown that MF significantly suppressed the growth and metastasis of EC cells in a dose-dependent manner, and promoted the cell apoptosis of EC [171]. 

*Gonadotropin-releasing hormone agonist (GnRH-a).* In patients with AEH or EC who are insensitive to oral progestin therapy, treatment with a combination of progestin and GnRH-a has a better treatment effect and a lower relapse rate [172]. In addition, GnRH-a in combination with letrozole can treat inoperable EC patients. GnRH-a reduces estrogen synthesis in the gonads, while letrozole inhibits estrogen synthesis in peripheral tissues; therefore, coadministration of letrozole and GnRH-a significantly downregulates estrogen levels in premenopausal women [173]. However, the GnRH-a regimen can lead to low pregnancy rates and reduced bone mass, which limits the widespread use of GnRH-a [174].

*Aromatase inhibitor (AI).* In premenopausal women, the development of EC is associated with elevated levels of androgen-to-estrogen conversion in adipose tissue and insufficient progesterone production due to decreased ovarian function. AI can reduce ER-mediated growth in EC by inhibiting estrogen synthesis in peripheral tissues [175]. Therefore, AI can be used as an adjunct to oral progestins. Straubhar et al. reported three cases in which each patient initially did not respond to progestin therapy, but the treatment effect improved after AI was combined with progestin therapy [175]. This suggests that obese women with EH or low-grade EC who wish to preserve their fertility could be treated with a combination of progestin and AI. 

*Androgen.* Studies have shown that androgens can increase PR expression in EC; therefore, exogenous androgen therapy may be an innovative therapy for EC patients with progestin resistance [176]. Progestin can also bind to AR and subsequently mediate the inhibition of EC cell proliferation [177]. Furthermore, high AR expression is associated with low-grade EC and favorable prognosis indicators, such as reduced metastasis and reduced lymphatic invasion [178]. In conclusion, it is conceivable that androgens can be used to treat progestin-insensitive EC, and that AR might be a therapeutic target. 

*Fourth-generation progestins.* Progestins are classified as first- to fourth-generation drugs. Norethisterone, levonorgestrel, and desogestrel are examples of first-, second-, and third-generation progestins, respectively. The fourth-generation progestins have the unique advantage of suppressing the proliferation of EC cells that are resistant to previous generations of progestins, such as MPA. Katsuki et al. found that HEC-88nu cells did not react to MPA, but the fourth-generation drug dienogest could inhibit the proliferation of HEC-88nu cells [179]. As another fourth-generation drug, nomegestrol acetate (NOMAC) inhibited the proliferation of RL95-2 cells more significantly than that of MPA [180]. Moreover, NOMAC acts not only on type I EC cells, but also on type II EC cells. It was found that NOMAC inhibited type II EC cells more effectively than levonorgestrel and cyproterone acetate [181].

### 5.3. Cocktail Drug Administration

High-dose progestin treatment often leads to some inevitable side effects, such as edema and weight gain. Lower doses of progestin can be used in combination with other drugs to overcome progestin resistance while reducing side effects. We summarized the drugs currently used in combination with progestin to enhance progestin sensitivity, termed cocktail drug administration, and they are shown in Table 3.

*Metformin.* It is known that hyperinsulinemia increases the risk of EC. Metformin, which is frequently prescribed as the first-line treatment for type 2 diabetes, has anti-proliferative effects against EC with insulin resistance and abnormal glucose levels. Metformin enhances the sensitivity of EC to progestin by increasing PR expression [182]. The addition of metformin to conventional progestin therapy improves recurrence-free survival, and appears to be more effective in obese women and patients with AEH [183,184]. The long-term combined treatment of metformin and MPA can also achieve a high complete response rate of 97% and a live yield of 45% [185]. In addition, metformin can counteract the adverse effects of progestin treatment, such as weight gain and glucose intolerance [185].

*Antipsychotic drugs.* Antipsychotic agents, including chlorpromazine (CPZ) and thioridazine (THIO), exert anti-oncogenic and progestin-sensitizing effects in EC. It was found that low-dose CPZ (5 mM) pre-treatment could effectively upregulate PRB expression [186]. Additionally, CPZ downregulated IGF-1R expression and induced PI3K/AKT phosphorylation, thereby inhibiting the development of EC [186]. Another antipsychotic drug, THIO, combined with MPA, may suppress the development of EC and enhance progestin sensitivity by upregulating the PRB expression and downregulating the EGFR expression. Additionally, after receiving treatment with THIO combined with MPA in EC cell lines, the PI3K/AKT signal transduction pathway was suppressed [187]. 

*PI3K/AKT/mTOR Pathway Inhibitors.* PI3K/AKT/mTOR inhibitors reported so far in combination with progestin mainly include three main categories: mTOR inhibitors, PI3K inhibitors, and AKT inhibitors. In phase II studies, temsirolimus and everolimus both showed effectiveness as monotherapies for EC [190,191]. Ridaforolimus could also provide clinical benefits in patients with recurrent or metastatic EC [192]. In vitro data showed that mTOR inhibitors increased the expression of PR messenger RNA in EC [193]. PI3K inhibitors combined with progestin might also enhance the progestin sensitivity for EC patients [13]. PI3K inhibitor LY294002 inhibited AKT activation and increased PRB transcriptional activity, thus enhancing the ability of MPA to inhibit cell proliferation and cause apoptosis in the EC cells [152]. In endometrial stromal cells, AKT inhibitors raised total and nuclear levels of PR [188]. MK-2206, an oral allosteric inhibitor of AKT, was found to reduce cell viability and enhance the death of EC cells [188]. Furthermore, MK-2206 increased PRB protein levels, regulated the expression of progestin-related genes, and worked together with progestin to reduce xenograft tumor volume. An AKT inhibitor, API-59CJ-OMe, likewise markedly raised PR levels in Ishikawa cells [189]. Therefore, further development of PI3K/AKT/mTOR inhibitors is necessary.

*Epigenetic modulation*. Epigenetic events modulate chromatin conformation, and mainly include variations in DNA methylation and histone acetylation [194]. There is more and more evidence that epigenetic modulators can restore functional PR expression, and progestin combined with epigenetic modulators can make EC more responsive to progestin therapy. Histone deacetylases inhibitor (HDACi) is one kind of molecularly targeted drug that suppresses the development of cancer by increasing the transcription of tumor-suppressor genes, thus arresting the cell cycle and inducing apoptosis [194]. Treatment with an HDACi restored the mRNA and protein expression of PR in EC cell lines [195]. HDACi LBH589 treatment led to cell cycle arrest in the G1 phase, which was further enhanced by progestin [196]. DNA methylation also affects PR expression. In Hec50co cells, the methylation of the PR promoter was reduced, and functional PR expression was restored by 5-aza-deoxycytidine [197]. Increased PR mRNA and protein expression can be attained by epigenetic modulation in EC cell lines, either with treatment with a DNA methyl transferase inhibitor (DNMTi) or with the combination of a DNMTi and an HDACi [195,198]. Additionally, EZH2 is a catalytic subunit of polycomb repressive complex 2, and leads to the trimethylation of histone H3, thus silencing the PR expression [199]. It was found that EZH2-specific inhibitors reduced EC cell proliferation and invasion, and inhibited tumor growth in mouse models [199].

### 5.4. Cytokines in Embryonic Microenvironment

The embryonic microenvironment contains a variety of protein factors, RNA and DNA, which may have the ability to reverse the malignant behavior of cancer cells [200]. Mintz et al. demonstrated that the tumorigenic phenotype of teratoma cells was reduced when cultured in the microenvironment of mouse embryonic blastocysts [200]. Sun et al. discovered that utilizing fluid from the embryonic sac, collected during in vitro fertilization (IVF), may reverse the progestin resistance of endometrial CSCs [95]. They found that placental alkaline phosphatase (ALPP), a protein factor released into the embryonic milieu, dramatically reversed progestin resistance by promoting endometrial CSC differentiation and downregulating the stemness genes NANOG, OCT4, and SOX2. In conclusion, cytokines in the embryonic microenvironment are safe, and might represent a new type of strategy for EC patients with progestin resistance.

### 5.5. Stem Cell Therapy Strategy

Stem cells have an important role in the regeneration and repair of the endometrium. It is reported that defective endometrial stromal fibroblasts (EMSFs) result in uterine factor infertility, endometriosis, and EC [201]. The pathophysiology of type I EC is also driven by the resistance of EMSF to progestin [202]. Menstrual blood-derived stem cells (MenSCs) could be induced to produce induced pluripotent stem cells (iPSCs) [201]. Replacement of abnormal EMSFs with iPSCs is a novel treatment strategy for endometrial disease. In addition, Miyazaki et al. showed that the WNT/CTNNB1 pathway played a crucial role in regulating PR expression during iPSC development [203]. Human umbilical cord-derived mesenchymal stem cells (hUCMSCs) also work for the damage repair of endometrial tissue [204]. Extracellular vesicles derived from hUCMSCs transported miR-302a to localized areas of EC, thereby inhibiting EC progression [205]. Therefore, the use of adult stem cells for the treatment of EC with progestin resistance has a bright future.

## 6. Conclusions and Future Perspectives

Progestin has long been studied therapeutically in hormone-based therapy for EH and EC. In certain patients, especially those who retain PR expression, it can reverse hyperplasia, AEH, and some early EC, therefore increasing overall survival. However, response rates in advanced diseases are low, and recurrence is frequent. As a result, the mechanism of progestin resistance needs to be studied in depth. Vault RNA is a non-coding RNA present in the dome ribonucleoprotein complex [206]. It has long been thought to be associated with multidrug resistance, owing to the effect on enzymes related to drug metabolism [207]. Whether vault RNA affects progestin drug metabolism and thus regulates progestin resistance requires further study. Additionally, one study has reported that exosomes are involved in tumor formation and progression of EC [208]. Since exosomes work as a genetic exchange vector in the tumor cell microenvironment, whether exosomes are involved in progestin resistance is of value for research. In conclusion, improvements in clinical results will depend on a deeper comprehension of progestin and progestin resistance. The multi-omic approach will play an important role in finding new molecular markers and specific therapeutic targets of EC. Future research to improve progestin resistance will focus on the development of new drug combinations and molecular typing of EC to determine the best form of the drug combination.

## Figures and Tables

**Table 1:** The markers of progestin resistance.

Classification	Marker	Function and Role	References
Cell proliferation	PI3K/AKT/mTOR	Blocking the PI3K/AKT/mTOR pathway induces autophagy and makes progestin-resistant cells more sensitive to progestin.	[99,100,101,102]
	PTEN	PTEN inhibits the PI3K/AKT/mTOR pathway by dephosphorylating related molecules and decreasing mTOR downstream activity.	[103,104,105,106]
	GRP78	GRP78, as an upstream gene of the PI3K/AKT pathway, indicated a poor response to progestin treatment in CAH samples.	[107,108,109]
	MSX1	MSX1 is reported to control the cell cycle, and its knockdown increased the effectiveness of progestin treatment in progestin-resistant EC cells.	[110,111]
Oxidative stress	Nrf2	Nrf2 activates genes that protect cells from intrinsic or exogenous oxidative stress and contributes to progestin resistance of EH and EC.	[112,113,114,115,116]
	AKR1C1	Overexpression of AKR1C1 may cause increased progestin catabolism, and AKR1C1 facilitates Nrf2-driven progestin resistance as a target gene of Nrf2.	[117,118]
	Survivin	Survivin is an inhibitor of apoptosis proteins; it regulates cell division, apoptosis, and angiogenesis, and mediates progestin resistance in EC.	[119]
	LASS2	LASS2 is a synthesizer of ceramides with broad tissue distribution. As the target gene of Nrf2, down-regulated LASS2 can increase the progestin sensitivity of EC.	[120]
Metabolism	GloI	GloI is a component of the glyoxalase system, and metformin reverses progestin resistance by downregulating the expression of GloI in EC.	[121,122,123]
	EGFR	EGFR promotes tumor growth and metastasis by increasing the PI3K/AKT signal and suppressing PRB, therefore causing EC to be insensitive to progestin.	[124,125,126,127]
	SIRT1	SIRT1 regulates cell proliferation, inflammation, and metabolism; SIRT1 is highly expressed in EC, especially in progestin-resistant EC.	[88,128,129,130,131]
	DHCR24	DHCR24 is an enzyme that mediates cholesterol synthesis. It is highly expressed in EC and is associated with progestin resistance.	[91,132,133]
	IGF	Both IGF-I and IGF-II suppress the expression of PR. IGF-II enhances cell proliferation through raising phosphorylation of AKT and p70S6K in EC.	[90,134,135]
Apoptosis	Fas/FasL	Fas-mediated apoptosis is critical for the endometrial cycle, and Fas/FasL dysregulation may play a role in the formation of progestin-resistant cells.	[70,136,137]
	Bcl-2	Bcl-2 prevents cells from entering apoptosis, and the expression of Bcl-2 in stromal cells can distinguish progestin responders from non-responders.	[27,71,138,139]
	PDCD4	PDCD4 is a target gene of progestin treatment in EC. Progestin inhibits the protein expression of PDCD4 via the PI3K/AKT signal pathway.	[140,141,142]
Nucleic acid regulation	MicroRNA	Five miRNAs limited the effect of progestin therapy in EC cells, and miR-96 reported to have the most obvious inhibitory effect on PR expression.	[59,143,144]
	HOTAIR	HOTAIR is a well-known lncRNA that suppresses PRB expression, and HOTAIR knockdown increased PRB transcription by recruiting LSD1 to the PRB promoter.	[145,146]
	DACH1	DACH1 plays a tumor-suppressing role in EC. DACH1 is positively associated with PR, and DACH1 knockdown enhanced progestin resistance.	[77,147,148,149]
	ARID1A	ARID1A deletion increased MPA resistance in EC by over-activating the PI3K/AKT signaling pathway and downregulating the PRB expression.	[150,151,152,153]
	HAND2	HAND2 suppresses estrogen-mediated signals in EC. Furthermore, methylation levels of HAND2 can predict patients’ response to progestin treatment.	[50,51]

**Table 2:** Overview of current clinical trials investigating novel progestin therapeutic strategies in EH and EC (2015–2022).

Register number	Conditions	Interventions	Phase	Number of Patients	Group
**Drug combination**					
NCT03077698	Endometrial Cancer	Drug: Sodium Cridanimod Drug: Progestin therapy	II	25	Sodium Cridanimod + Progestin therapy
NCT02064725	Recurrent or Persistent Endometrial Carcinoma	Drug: Sodium cridanimod	II	8	Sodium cridanimod + megestrol acetate or MPA
NCT04792749	Endometrial Cancer Stage I	Drug: Metformin	III	77	Metformin + MPA
NCT05316935	Endometrial Neoplasms Atypical Endometrial Hyperplasia Progesterone Resistance	Drug: GnRHa Drug: Letrozole 2.5 mg Drug: Diane-35 Drug: Metformin	II–III	80	GnRHa + letrozole vs. Ethinylestradiol cyproterone + metformin
NCT04046185	Endometrial Cancer Stage I	Drug: PD-1 inhibitor Drug: Progesterone	I	60	PD-1 inhibitor + progesterone
NCT04607252	Atypical Endometrial Hyperplasia	Drug: Metformin plus megestrol acetate Drug: Megestrol Acetate	II–III	12	Metformin + megestrol acetate
**Application of LNG-IUS**					
NCT02990728	Endometrial Cancer	Drug: Metformin Device: Mirena	II	120	Metformin + Mirena
NCT03463252	Endometrial Cancer Atypical Endometrial Hyperplasia	Drug: Progesterone Device: Mirena Drug: GnRH agonist	II–III	224	MPA + Mirena vs. progesterone GnRH-a + Mirena vs. Mirena
NCT01074892	Endometrial Hyperplasia	Drug: Provera (medroxyprogesterone/progestin) Device: Mirena (levonorgestrel)	IV	170	medroxyprogesterone/progestin vs. Mirena (levonorgestrel)
NCT04385667	Atypical Endometrial Hyperplasia	Device: Levonorgestrel intrauterine system (LNG-IUD) Drug: Oral megesterol 160 mg daily	II–III	140	Oral megesterol vs. LNG-IUS
NCT04897217	Endometrial Hyperplasia	Drug: Megestrol acetate Drug: Levonorgestrel drug implant	III	40	Megestrol acetate vs. LNG-IUS
NCT03992937	Endometrial Hyperplasia Without Atypia	Drug: Vaginal micronized progesterone Device: Levonorgestrel-intrauterine system	Not Applicable	132	Vaginal micronized progesterone vs. LNG-IUS
**In combination with radiotherapy**					
NCT05255653 (NSMP-ORANGE trial)	Endometrial Cancer	Radiation: Pelvic external beam radiotherapy Drug: Medroxyprogesterone acetate Drug: Megestrol acetate Other: Observation	III	1611	Pelvic external beam radiotherapy + oral progestagens
**In combination with chemotherapy**					
NCT00739830	Endometrial Cancer	Drug: Ridaforolimus Drug: Medroxyprogesterone acetate tablets OR megestrol acetate Drug: Chemotherapy	II	130	Ridaforolimus vs. MPA or megestrol acetate + chemotherapy
**In combination with surgery**					
NCT04008563	Endometrial Cancer Atypical Hyperplasia Bariatric Surgery Candidate	Bariatric surgery	Not Applicable	36	Bariatric surgery + progestin intrauterine device
NCT04362046	Endometrial Hyperplasia Endometrial Cancer Gynecologic Cancer	Procedure: Hysteroscopic uterine resection	Not Applicable	30	Progestin + hysteroscopic uterine resection

**Table 3:** Cocktail drug administration to reverse progestin resistance.

Drug Class	Agent	Function	References
**Hypoglycemia agent**	Metformin	Metformin enhanced the progestin sensitivity of EC by decreasing GloI expression and downregulating Nrf2 and survivin expression.	[182,183,184,185]
**Antipsychotic drugs**	Chlorpromazine	CPZ pretreatment may increase PRB expression and CPZ, phosphorylate PI3K/AKT, and downregulate the expression of IGF-IR.	[186]
	Thioridazine	THIO combined with MPA inhibited the PI3K/AKT/mTOR pathway and enhanced progestin sensitivity by downregulating EGFR and upregulating PRB.	[187]
**PI3K/AKT/mTOR pathway inhibitors**	PI3K Inhibitors	Pretreatment with the PI3K inhibitor LY294002 caused cell apoptosis, increased PRB expression, and, therefore, enhanced the therapeutic effect of MPA in EC.	[13,152]
	AKT Inhibitors	MK-2206, an active AKT inhibitor, regulated the expression of progestin-related genes, increased PRB protein levels, and induced apoptosis of EC cells.	[188,189]
	mTOR Inhibitors	mTOR Inhibitors increased the expression of PR messenger RNA in patients with recurrent or metastatic EC, and suppressed the growth of EC.	[190,191,192,193]
**Epigenetic modulation**	Histone deacetylating inhibitors	HDACi therapy restored the protein and mRNA expression of PR in EC cell lines. LBH589 treatment resulted in cell cycle arrest in G1, which was further promoted by progestin.	[194,195,196]
	DNA methyl transferase inhibitor	5-aza-deoxycytidine, as one kind of DNMTi, reduced the methylation of the PR promoter and restored functional PR expression in EC cells.	[197,198]
	Histone methylation inhibitors	EZH2 caused trimethylation of histone H3, thus silencing the PR expression. EZH2-specific inhibitors reduced EC cell proliferation and invasion.	[199]

## Abbreviations

EC: endometrial cancer; EH: endometrial hyperplasia; AEH: atypical endometrial hyperplasia; EIN: endometrioid intraepithelial neoplasia; MPA: medroxyprogesterone acetate; MA: megestrol acetate; LNG-IUS: levonorgestrel intrauterine system; PR: progestin receptor; PRE: progestin response element; GPR30: G protein-coupled receptor 30; VEGF: Vascular endothelial growth factor; bFGF: basic fibroblast growth factor; FasL: Fas ligand; FADD: Fas associated death domain; CAH: complex atypical hyperplasia; EMT: epithelium-mesenchymal transition; ER: estrogen receptor; 17-HSDs:17β-hydroxysteroid dehydrogenases; AR: androgen receptor; fibroblast growth factor: FGF; PTMs: Posttranslational modifications; EGFR: epidermal growth factor receptor; EGFR-TK: EGFR tyrosine kinase; SHBG: sex hormone-binding globulin; GPER: G-protein-coupled estrogen receptor; ADAMTS: A disintegrin and metalloproteinase with thrombospondin motifs; IGF: insulin-like growth factors; CSCs: Endometrial cancer stem cells; GloI: Glyoxalase I; SIRT1: Sirtuin 1; IGF-IR: IGF-I receptor; HOTAIR: HOX transcript antisense intergenic RNA; MF: Mifepristone; TCGA: the cancer genome atlas; MSI: microsatellite instability; MMR: mismatch repair; GnRH-a: Gonadotropin-releasing hormone agonist; AI: Aromatase inhibitor; NOMAC: nomegestrol acetate; CPZ: chlorpromazine; THIO: thioridazine; HDACi: Histone deacetylases inhibitor; DNMTi: DNA methyl transferase inhibitor; EMSFs: endometrial stromal fibroblasts; MenSCs: Menstrual blood-derived stem cells; iPSCs: induced pluripotent stem cells; hUCMSCs: human umbilical cord derived mesenchymal stem cells.
